# Persistent Effect Test and Internal Microscopic Monitoring for PEM Water Electrolyzer

**DOI:** 10.3390/mi12050494

**Published:** 2021-04-27

**Authors:** Chi-Yuan Lee, Chia-Hung Chen, Guo-Bin Jung, Yu-Xiang Zheng, Yi-Cheng Liu

**Affiliations:** 1 Yuan Ze Fuel Cell Center, Department of Mechanical Engineering, Yuan Ze University, Taoyuan 32003, Taiwan; guobin@saturn.yzu.edu.tw (G.-B.J.); f129771057f@gmail.com (Y.-X.Z.); pierce0922@gmail.com (Y.-C.L.); 2 HOMYTECH Global Co., Ltd., Taoyuan 33464, Taiwan; chenjahon@gmail.com

**Keywords:** PEM water electrolysis cell stack, accelerated aging, flexible integrated microsensor, microscopic diagnosis

## Abstract

As the environmental considerations rise all over the world and under the drive of renewable energy policy, the society of hydrogen energy will come out gradually in the future. The proton exchange membrane water electrolyzer (PEMWE) is a very good hydrogen generator, characterized by low cost, high efficiency and zero emission of greenhouse gases. In this study, the micro temperature, humidity, flow, pressure, voltage, and current sensors were successfully integrated on a 50 μm thick Polyimide (PI) substrate by using micro-electro-mechanical systems (MEMS) technology. After the optimal design and process optimization of the flexible 6-in-1 microsensor, it was embedded in the PEMWE for a 500-h persistent effect test and internal real-time microscopic monitoring.

## 1. Introduction

In recent years, various countries have been paying attention to the issue of environmental protection, relieving climate change is the main challenge to the energy policies of the developed countries. At the present stage, human civilization severely depends on fossil energy, which releases greenhouse gases and other pollutants, it is the major factor in global warming [1,2]. During 1971~2020, fossil fuel was in the majority of the fuels for electricity generation. In 2019, the low-carbon electricity exceeded coal, but the IEA (International Energy Agency) still emphasized that low-carbon energy should be expanded greatly [3].

In 2008, the world went through an economic tsunami while the oil price rose high (Hirscher, 2009), maintaining and expanding prosperous civilization need a considerable energy supply, depending on a few oil-exporting countries will bring enormous risks to human economy. According to the current consumption levels, fossil fuel will remain at a high price. The present fossil fuel reserves can supply petroleum for at most 40 years, natural gas for 60 years, and coal for 156 years [4]. Shortages will come soon if the consumption is not reduced [2,3,4,5,6,7,8]. The shale oil regarded as the petroleum substitute may result in water pollution, high mining cost, high technical difficulty, and high energy consumption problems [9]. Using renewable natural energy resource flow to power the society, separating from the exhausting energy reserves is the current target of the energy circle in the established policy scenario of sustainable development [7].

Taiwan has listed green energy technology as one of the “5 + 2” industrial innovation programs, the photovoltaic and wind power generations are the most critical development projects, aiming to make renewable energy account for 20% of power generation in 2025. For wind power generation, the Ministry of Economic Affairs has formulated a “4-Year Promotion Plan for Wind Power Generation”, planning to push the foundation of wind power generation and promote energy diversification and autonomous supply of electricity [10]. However, solar energy or wind energy has a fatal problem, i.e., unstable power supply. Therefore, energy storage devices will play a leading part. The lithium battery extensively used in current energy storage devices has several problems. The lithium used in the lithium battery is a scarce resource, a high consumption has a high cost, it is unsuitable for large energy storage devices. The leakage of electrolyte may induce combustion, even explosion. Hydrogen energy is one of the optimal energy storage systems of green energy [11]. The proton exchange membrane (PEM) water electrolysis cell stack has many advantages, such as less corrosivity, it can be operated at a lower voltage, higher current density and higher temperature and pressure, so as to increase efficiency (80–90%). In response to these policies and trends, this research intends to develop a microsensor for real-time wireless monitoring of PEMWE. After improving the flow channel of PEMWE, the performance of PEMWE will be increased, which can be used in homes, street lights, streets, etc. Solar power storage systems and even hydrogen exchange stations for hydrogen energy transportation vehicles of PEM fuel cells are rushing to attack Taiwan’s hydrogen energy market.

Müller et al. [12] studied different temperatures (60 °C, 70 °C, 80 °C, 90 °C) and drag coefficients (1.0, 1.5, 2.0, 2.5) and the transmission relationship between Nafion membrane thickness and water spreading. Fujimura et al. [13] studied the influence of surface wettability on hydrogen evolution reaction (HER) activity and efficiency. Saccardo et al. [14] attached different cations to the Nafion117-MEA (Membrane Electrode Assembly) membrane material surface, the experiment proved that humidity could influence the performance of MEA membrane material. Möckl et al. [15] built an internal heat transfer model of PEMWE to perform experiments on heat transfer and flow control. The experiments proved that flow control could effectively control the temperature difference between the flow channel inlet and outlet in a specific range, preventing the MEA overheating failure. Ferrero et al. [16] studied the performance difference in PEM water electrolyzer with different water flows, and found that high pressure resulted in higher performance, and low pressure resulted in lower performance. Grigoriev et al. [17] operated the PEMWE at a constant temperature of 85 °C and different pressures, and found that the energy consumption of water electrolysis could be reduced effectively by increasing pressure appropriately. Chakik et al. [18] tried different voltage conditions to find out the optimal conditions for producing hydrogen. Trinke et al. [19] tested the influence of the current density on the oxygen flux in the cathode outlet with and without Pt catalyst. Sartory et al. [20] operated a water electrolyzer in different working conditions, the result showed that the higher the temperature was, the higher was the hydrogen production efficiency.

Responding to these policy trends and the bottleneck of internal diagnosis of PEMWE, this study uses MEMS technology to integrate micro voltage, current, temperature, humidity, flow, and pressure sensors on a 50 μm thick PI substrate, after the optimal design and process optimization of flexible 6-in-1 microsensor, it is embedded in the PEMWE for 500-h real-time microscopic monitoring of internal persistent effect.

## 2. Sensing Principle of Flexible 6-in-1 Microsensor

### 2.1. Micro Temperature Sensor

**Figure 1:**
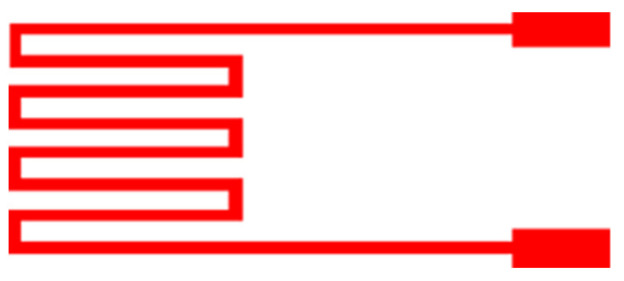
Schematic diagram of resistive micro temperature sensor.

This study uses the resistive temperature sensing principle. In a certain temperature range, the resistance of metal changes due to temperature change. The sensor structure is shown in Figure 1. The Au is used as temperature-sensitive resistor material for stable chemical properties, simple process, and high linearity. The relation can be reduced to Equation (1).

*R*_t_ ≈ *R*_0_ (1 + *α* × Δ*T*) (1)

where *R_t_* is the resistance (Ω) at t °C; *R*_0_ is the resistance (Ω) at 0 °C; *α* is the TCR (%/°C); Δ*T* is the temperature difference (°C) [21].

### 2.2. Micro Humidity Sensor

**Figure 2:**
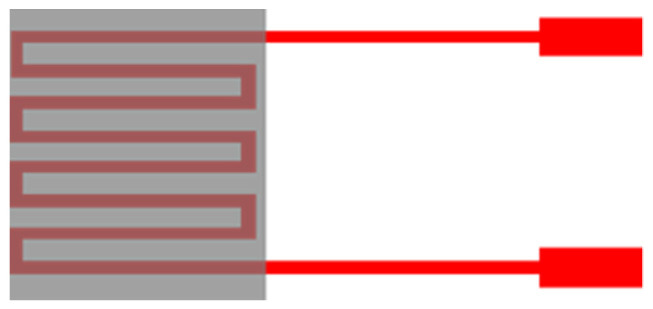
Schematic diagram of resistive micro humidity sensor.

The micro humidity sensor uses PI 9305 as humidity sensing material, this material must be nonconducting in general. When the volume of the material increases with moisture pickup, the adhesive circuit resistance increases [22], as shown in Figure 2.

### 2.3. Micro Flow Sensor

**Figure 3:**
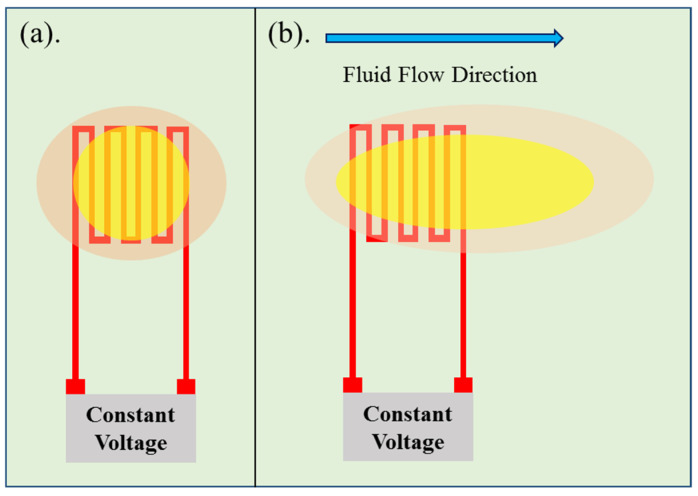
Schematic diagram of hot-wire micro flow sensor principle. (**a**) the flow is zero; (**b**) the flow is not zero.

The hot-wire micro flow sensor principle is that constant voltage is imported into the resistance heater to generate a heat source, as the heat carried away by the fluid flow increases, the resistance value of resistance heater decreases accordingly. The hot-wire micro flow sensor principle is shown in Figure 3. According to the King’s law, the relationship between the heat dissipation rate and fluid flow rate is expressed as Equation (2). When the flow is not zero, Equation (2) can be changed to Equation (3) [23].

*Q* = *I*^2^ × *R* = *I* × *V* = (*A* + *B* × *U^n^*) (*T_s_* − *T_o_*) (2)

*Q* = (*A* + *B* × *U*^0.5^) Δ*T* (3)

where *Q* is the electric power from the external power supply, *I* is the electric current through hot wire, *R* is the resistance of hot wire, *A* is the heat transfer coefficient of fluid, *U* is the flow rate of fluid, *n* is the coefficient of correlation between heat *Q* and flow rate *U*.

### 2.4. Micro Pressure Sensor

The general capacitive pressure sensor is two parallel interlayered electrodes, sandwiching a nonconducting dielectric layer to form a sandwich structure. The computing equation for the capacitance value between two parallel electrodes is Equation (4) [23].

(4) $$\Delta C=\varepsilon_{r}\varepsilon_{0}\frac{A}{\Delta d}$$

where *ε*_0_ is the constant 8.854 × 10^−12^ (F/m), *ε_r_* is the dielectric constant of material, *A* is the projection overlapping area of two parallel electrodes, Δ*d* is the rate of change in distance between two electrodes.

**Figure 4:**
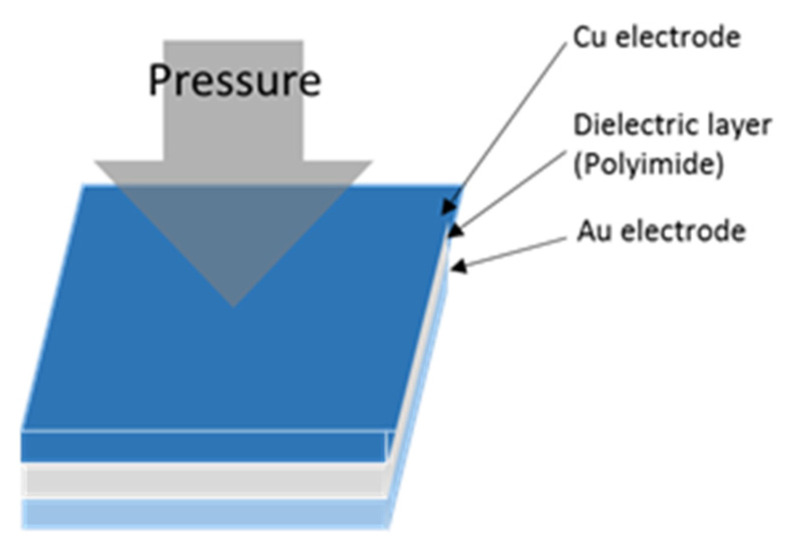
Schematic diagram of micro pressure sensor.

The commercially available pressure sensor is a hollow structure, when it receives pressure, the thin film deformation is nonlinear, leading to poor sensor linearity and sensitivity. To solve the aforesaid problem, this study uses Fujifilm Durimide^®^ PI 9305 (FUJIFILM Electronic Materials Taiwan Co., Ltd., Hsin-Chu, Taiwan) with a high dielectric constant and small E-modulus as a dielectric layer. When the solid dielectric layer is pressed, the stress and deformation are averaged, relatively matching linear Equation (4), the schematic diagram of a micro pressure sensor is shown in Figure 4.

### 2.5. Micro Voltage Sensor

**Figure 5:**
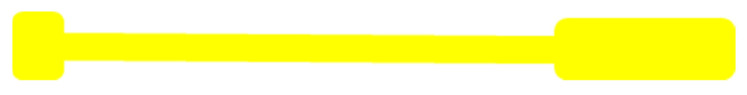
Schematic diagram of micro voltage sensor.

This study uses a microminiaturized metal probe as a micro voltage sensor. The sensing area of a micro voltage sensor and the link circuit board at the tail end of the sensor are exposed, the other part is insulated by the insulating layer of PI 9320, so as to detect the voltage in local region [23], as shown in Figure 5.

### 2.6. Micro Current Sensor

**Figure 6:**
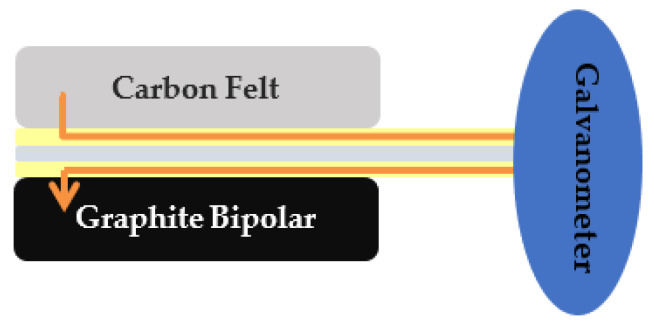
Schematic diagram of micro current sensor operating principle.

The same as the micro voltage sensor principle, this study uses a microminiaturized metal probe as micro current sensor, only detecting the current in local region [22], as shown in Figure 6.

## 3. Process Development of Flexible 6-in-1 Microsensor

The development of a flexible 6-in-1 microsensor for microscopic diagnosis inside water electrolyzer includes selecting a feasible sensing principle, designing the sensor pattern and using appropriate process material, so as to obtain accurate internal real-time microscopic diagnosis information in the electrochemical environment of water electrolyzer.

**Figure 7:**
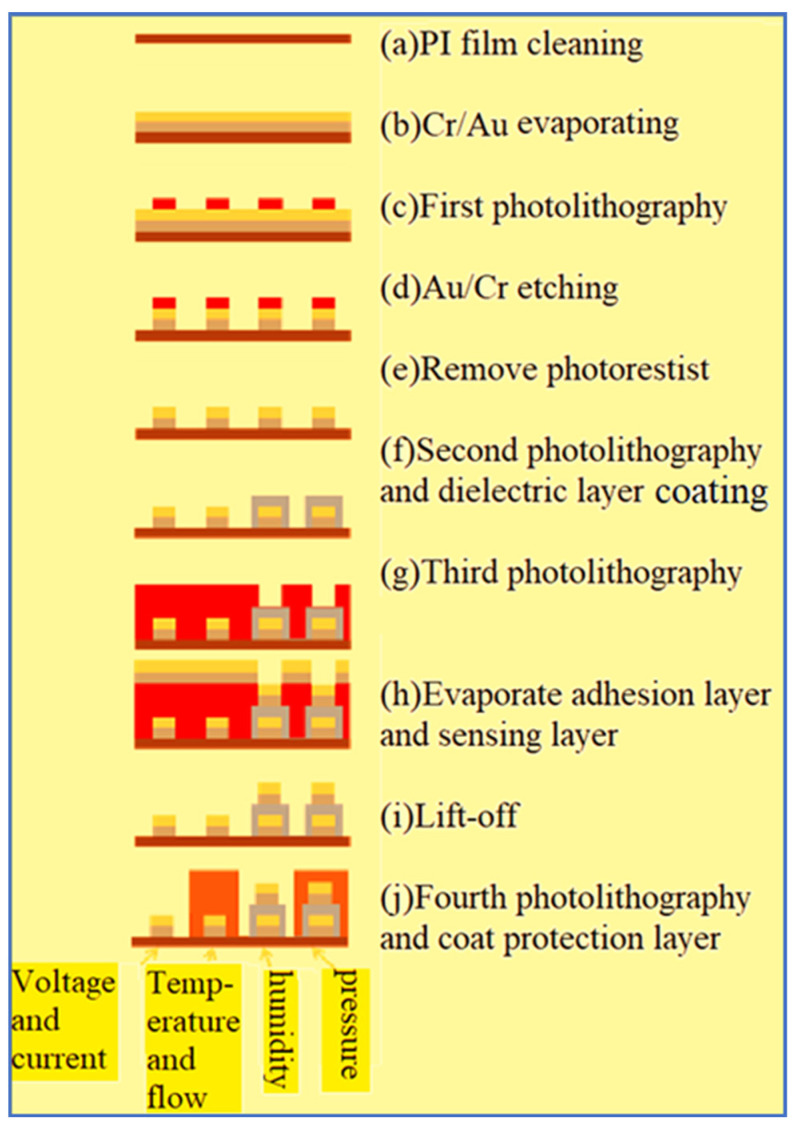
Process diagram of flexible 6-in-1 microsensor.

In terms of process, surface micromachining technology is used, including deposition, lithography, wet etching, and metal lift-off. The fabrication process is shown in Figure 7.

(a) PI film cleaning and fixing

The PI film substrate (50 μm thick) is cleaned in organic solvent ethanol, and then put in the organic solvent acetone preheated to boiling, and the acetone is volatilized.

(b) Evaporate metal

The metal is evaporated by E-beam evaporator, the 300 Å thick Cr and 1500 Å Au are deposited at a deposition rate of 1 Å/s.

(c) First photolithography

The positive photoresist (AZ^®^ P4620, Microchemicals GmbH, Ulm, Germany) is uniformly coated on the sample by spin coater. The pattern of integrated microsensor is defined by photolithography.

(d) Metal etching

After the pattern is transferred to the positive photoresist (AZ^®^ P4620), the pattern is retransferred to the Cr or Ti and Au metal film by wet etching. The unnecessary Au/Cr is removed by using commercial Type-TFA Au etching solution and Cr-7T Cr etching solution.

(e) Remove photoresist

The photoresist is removed by acetone.

(f) Second photolithography and coating dielectric layer

The PI 9305 is used as the material of the dielectric layer. Therefore, the purpose of the secondary exposure and development is to define and complete the dielectric layer of the micro humidity sensor.

(g) Third photolithography and metal lift-off

This study uses two photoresists as the sacrificial layer of metal lift-off. The first one is negative photoresist APOL-3202 (M&R Nano Technology Co. Ltd., Yangmei, Taiwan) its minimum line width can be 2 μm, there will be slight expansion after photolithography that will be very helpful to the subsequent evaporation process, because the evaporated metal cannot completely cover the photoresist, favorable for the photoresist to contact the remover, guaranteeing the completion of metal lift-off.

(h) Evaporate adhesion layer and sensing layer

E-beam evaporator evaporates chromium and gold as the sensing layer and electrode layer.

(i) Metal lift-off

Remove the remaining photoresist, and the metal covering it will be lifted off. The remaining metal will form the desired circuit pattern.

(j) Fourth photolithography and coating protection layer

**Figure 8:**
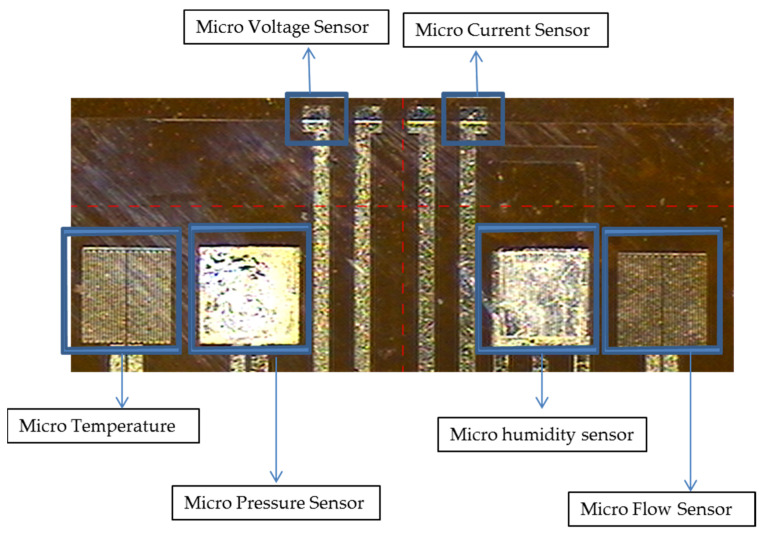
Optical micrograph of 6-in-1 microsensor.

To avoid the flexible 6-in-1 microsensor being destroyed by the closing pressure of end plate inside the PEM water electrolyzer, an insulation protection layer adapted to a highly chemical environment with high mechanical strength must be used. On the other hand, the protection layer can avoid short circuit of the flexible 6-in-1 microsensor and graphite plate. The protection layer must be pattern definable insulating material, the fundamental purpose is to expose the sensing area of micro voltage and current sensors to directly contact the flow channel rib, and to expose the signal pad for subsequent signal transfer and output. This study uses the PI (Fujifilm Durimide^®^ PI 9320, FUJIFILM Electronic Materials Taiwan Co., Ltd., Hsin-Chu, Taiwan) as the protection layer of the flexible 6-in-1 microsensor. The optical micrograph of flexible 6-in-1 microsensor is shown in Figure 8.

## 4. Flexible 6-in-1 Microsensor Reliability Correction

After the fabrication of the flexible 6-in-1 microsensor, the 6-in-1 microsensor shall be corrected for signal measurement and reliability validation. Three flexible 6-in-1 microsensors are corrected one by one, and embedded in the anode runner plate of the PEM water electrolyzer for internal real-time microscopic diagnosis after the reliability is confirmed, so as to guarantee the correctness of experiment data.

### 4.1. Micro Temperature Sensor Reliability Test (Temperature Correction)

**Figure 9:**
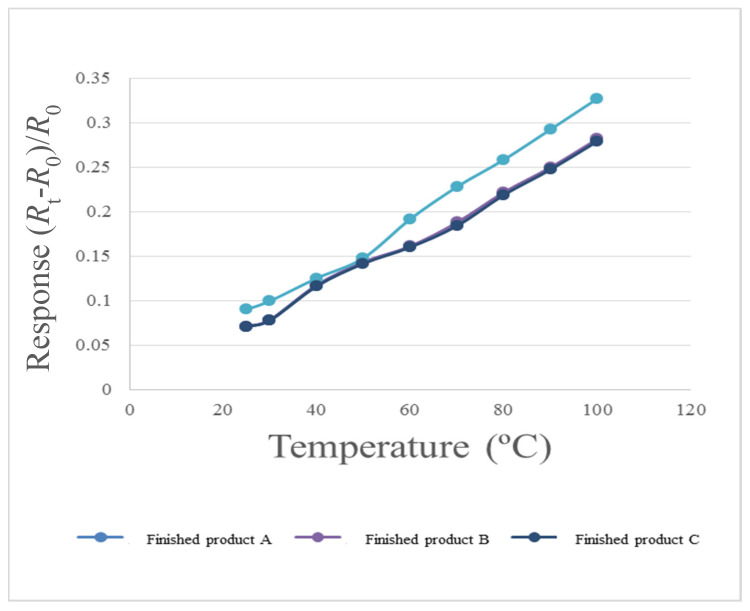
Correction curve of micro temperature sensor.

To make the correction environment closer to the practical situation, this study uses program controlled constant temperature and humidity testing machine (Hung ta HT-8045A Environmental Chamber, Hung Ta Instrument Co., Ltd., Taichung, Taiwan) as the reference of the correction environment. When the PEM water electrolyzer is in operation, the flow channel is full of DI water, so the humidity is fixed at 100% during temperature correction. The temperature correction range of micro temperature sensor is 25 °C to 100 °C, one signal is captured every 10 °C since 30 °C, nine signals are captured, as shown in Figure 9.

### 4.2. Micro Humidity Sensor Reliability Test (Relative Humidity Correction)

**Figure 10:**
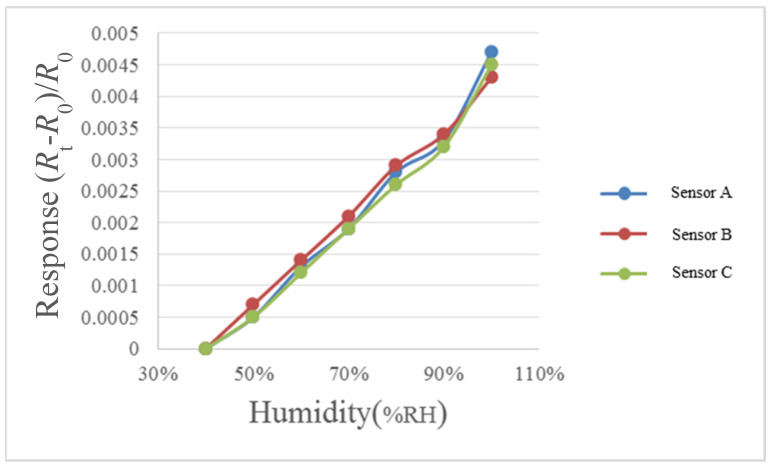
30 °C relative humidity correction curve diagram.

**Figure 11:**
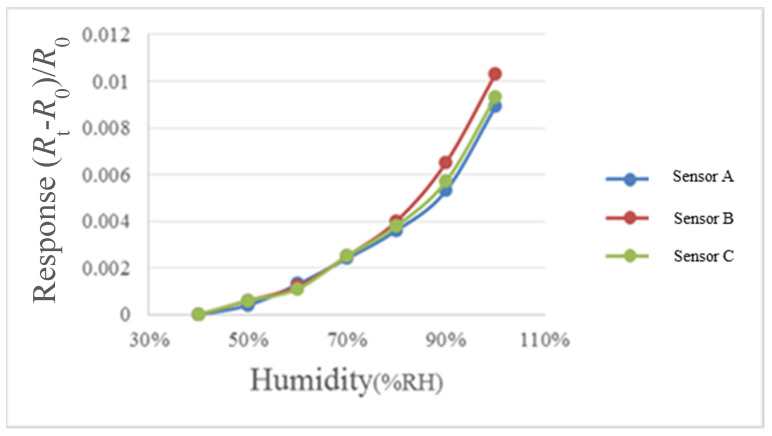
50 °C relative humidity correction curve diagram.

**Figure 12:**
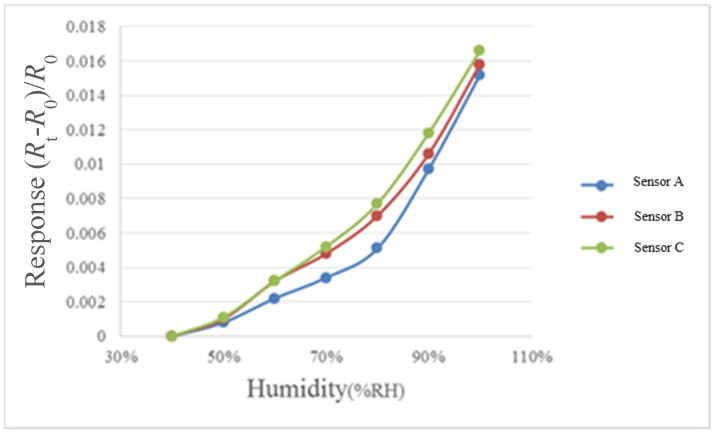
70 °C relative humidity correction curve diagram.

For humidity correction, the constant temperature and humidity testing machine is used as environmental criteria, in the operation conditions of relative humidity of 40% to 100% and fixed temperatures of 30 °C, 50 °C, and 70 °C, each time when the relative humidity is increased by 10%, one recording point is made, and the heater on the micro humidity sensor is heated to evaporate the residual moisture at previous recording point, each time after 30 minutes’ stabilization, the NI PXI cabinet data acquisition unit is used to extract the resistance value of micro humidity sensor instantly, so as to obtain the correction curve, as shown in Figure 10, Figure 11 and Figure 12.

### 4.3. Micro Flow Sensor Reliability Test (Flow Correction)

**Figure 13:**
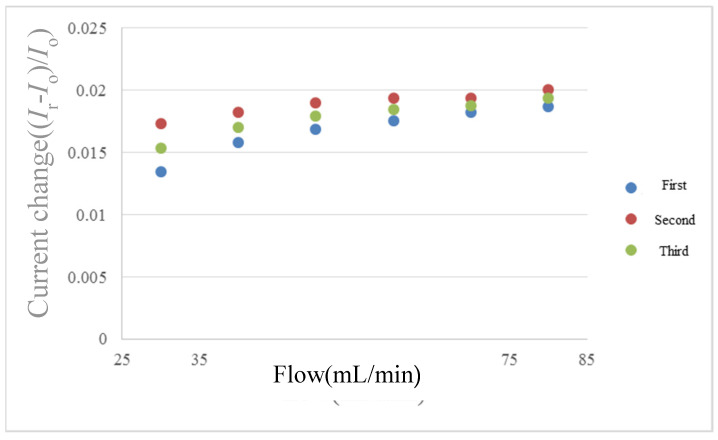
Micro flow sensor correction curve diagram.

The LEADFLUID BT100S-1 acid and alkali-resistant speed adjusting peristaltic pump provides steady flow for flow correction. The peristaltic pump uses peristaltic pipes for alternate extrusion and release to convey fluid. The advantage is that the peristaltic pipe is used as a pump chamber, the fluid only flows through the peristaltic pipe without contaminating the pump body, and the peristaltic pipe will not contaminate the fluid, it is a good device for transferring DI water. The flow range of LEADFLUID BT100S-1 is 30~1700 mL/min, and the flow accuracy is ±0.5%. The flow correction curve is shown in Figure 13.

### 4.4. Micro Pressure Sensor Reliability Test (Pressure Correction)

**Figure 14:**
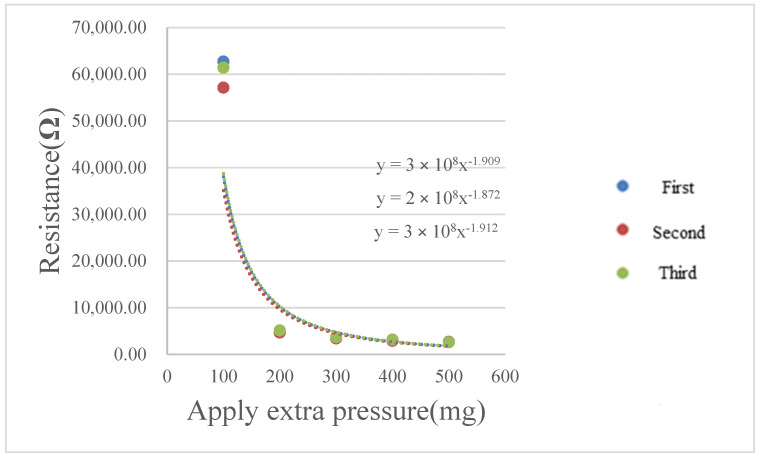
Micro pressure sensor correction curve diagram.

The rate of change in capacitance of the developed micro pressure sensor is too low, so the rate of change in the capacitance value cannot be measured. In order to know the local pressure change inside the water electrolyzer, the Force Sensitive Resistor 0.2 inch pressure sensor of Interlink Electronics is embedded in the water electrolyzer. As the commercial sensor has a thickness, the water electrolyzer has slight leakage. The commercial pressure sensor correction curve is shown in Figure 14.

## 5. Persistent Effect Test and Internal Microscopic Diagnosis for PEM Water Electrolyzer

**Figure 15:**
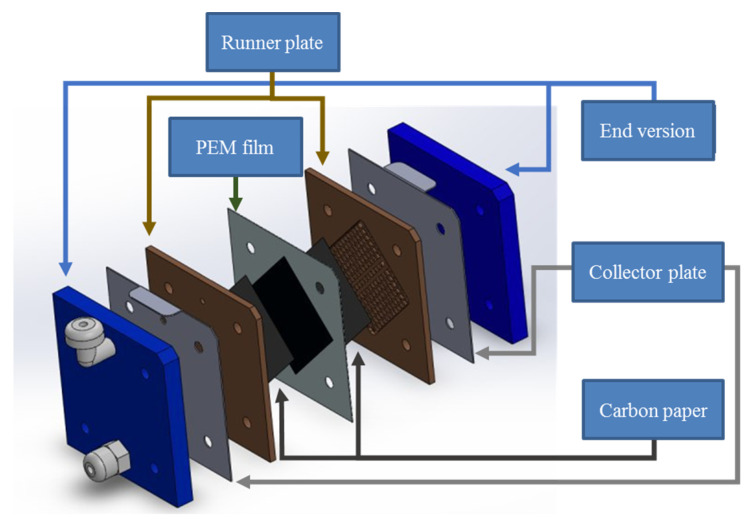
Exploded view of parts of proton exchange membrane (PEM) water electrolyzer.

This section will introduce the PEM water electrolyzer developed in cooperation with HOMY Technology, the model is EL9-9EM, including the structure of the PEM water electrolyzer, the selection of materials, and the design of the flow field. In addition, coupled with the collector plate designed in this study, refer to the assembly procedures of related technologies to complete the assembly of the PEM water electrolyzer. The schematic diagram is shown in Figure 15.

After the correction of the flexible 6-in-1 microsensor, the 500-h persistent effect test and internal microscopic diagnosis for PEM water electrolyzer are performed. In the conditions of constant current and constant voltage, the local voltage, current, temperature, humidity, and flow variations and distributions inside the PEM water electrolyzer are monitored instantly.

**Figure 16:**
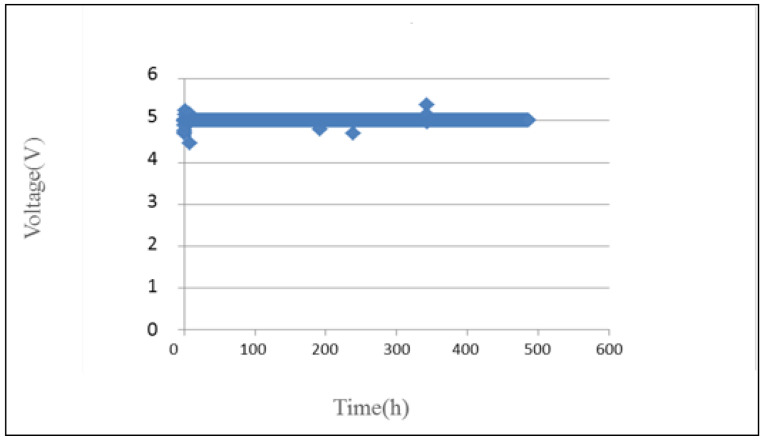
Curve of voltage inside PEM water electrolyzer.

In terms of the voltage distribution inside PEM water electrolyzer, the voltage curve is very stable in the 500-h power supply, as shown in Figure 16.

**Figure 17:**
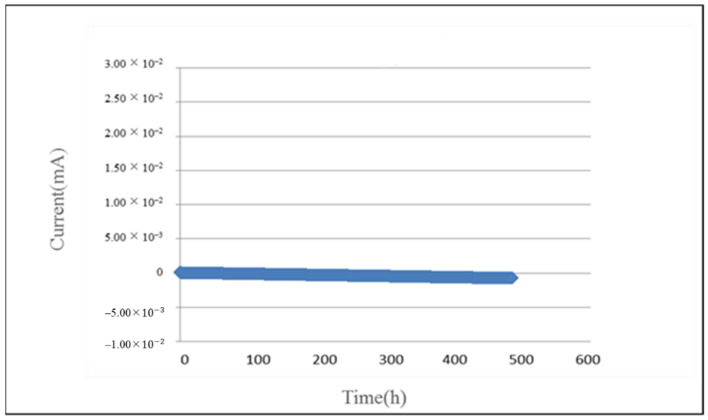
Curve of current inside PEM water electrolyzer.

The current of PEM water electrolyzer decreases from 0.15 mA to 0.12 mA in the 500-h operation test, as shown in Figure 17.

**Figure 18:**
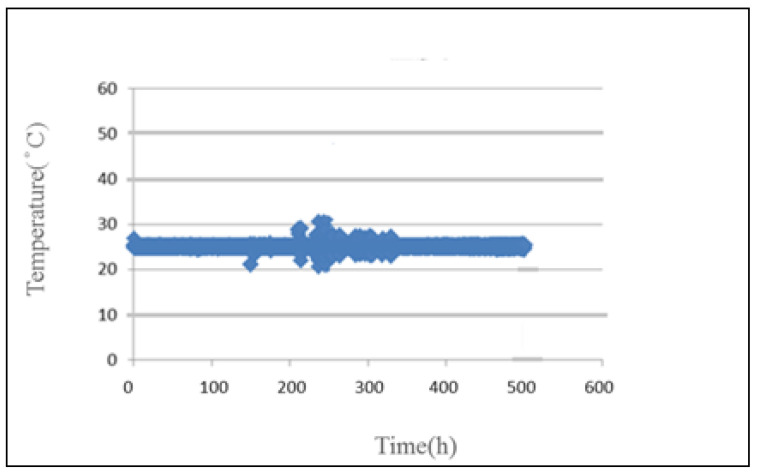
Curve of temperature inside PEM water electrolyzer.

The temperature distribution inside the PEM water electrolyzer is monitored at an operating temperature of 25 °C and constant current. As the water flows through the water electrolyzer continuously, the internal temperature remains at about 25 °C, there is no significant difference, as shown in Figure 18.

**Figure 19:**
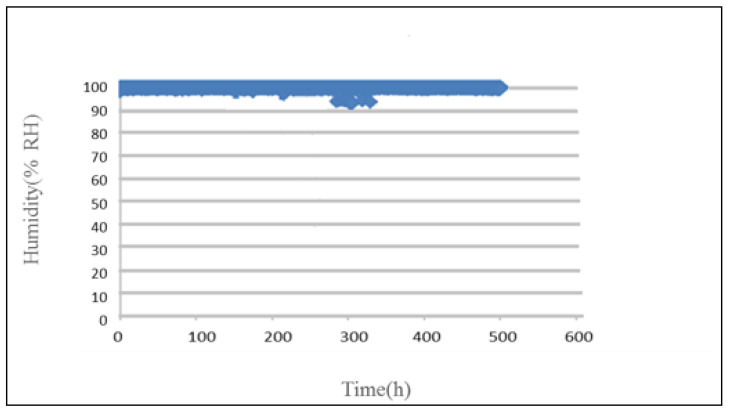
Curve of humidity inside PEM water electrolyzer.

As the PEM water electrolyzer shall be tested in pure water, the relative humidity is very close to 100%, as shown in Figure 19.

**Figure 20:**
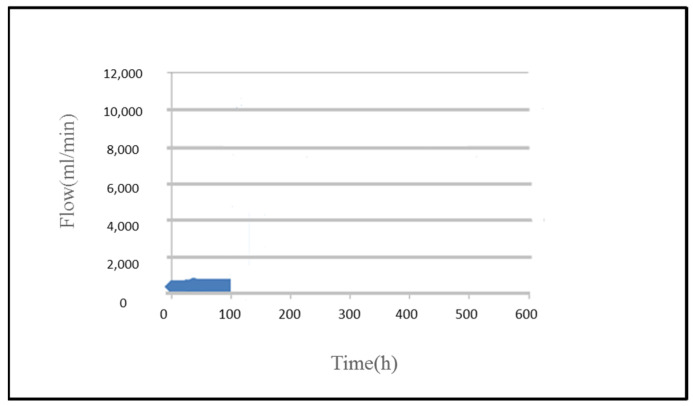
Curve of flow inside PEM water electrolyzer.

As the operating voltage is high (5V), the micro flow sensor is destroyed after 100 h, the flow inside PEM water electrolyzer cannot be monitored anymore, as shown in Figure 20.

## 6. Conclusions

This study uses MEMS technology to develop an electrochemical environment resistant flexible 6-in-1 microsensor, the micro voltage, current, temperature, humidity, flow and pressure sensors are successfully integrated on a 50 μm thick Polyimide (PI) film substrate, and the PI (Fujifilm Durimide^®^ PI 9320) resistant to the corrosion of electrochemical environment is used as a protection layer. This flexible 6-in-1 microsensor has six functions, as well as such advantages as corrosion resistance, small area, high sensitivity, good temperature tolerance, real-time measurement, and arbitrary placement.

Without influencing the operation of PEM water electrolyzer, two sets of flexible 6-in-1 sensors are successfully embedded in the anode runner plate, the temperature and flow are corrected, and the correction curves are highly linear. In the operation process of PEM water electrolyzer, the local voltage, current, temperature, humidity, and flow information inside the PEM water electrolyzer is extracted successfully by a data acquisition unit. Finally, the anode runner plate is tested at different temperatures and flows. The test result shows that the temperature and current are the key factors in PEM water electrolyzer.
